# Adalimumab therapy is associated with increased faecal short chain fatty acids in hidradenitis suppurativa

**DOI:** 10.1111/exd.14665

**Published:** 2022-08-30

**Authors:** Artiene Tatian, Sara Bordbar, Samuel Der Sarkissian, Jane A. Woods, Geoffrey D. Cains, Chun Wie Chong, Eliana Mariño, John W. Frew

**Affiliations:** ^1^ Department of Dermatology Liverpool Hospital Liverpool New South Wales Australia; ^2^ University of New South Wales Sydney New South Wales Australia; ^3^ Infection and Immunity Program, Department of Biochemistry, Biomedicine Discovery Institute Monash University Melbourne Victoria Australia; ^4^ School of Pharmacy Monash University Malaysia Selangor Malaysia; ^5^ Laboratory of Translational Cutaneous Medicine Ingham Institute of Applied Medical Research Liverpool New South Wales Australia

**Keywords:** adalimumab, hidradenitis suppurativa, microbiota, SCFAs, tunnels

## Abstract

Altered gut microbiota composition has been observed in individuals with hidradenitis suppurutiva (HS) and many other inflammatory diseases, including obesity, type 1 and type 2 diabetes. Here, we addressed whether adalimumab, a systemic anti‐inflammatory therapy, may impact the microbiota biochemical profile, particularly on beneficial metabolites such as short‐chain fatty acids (SCFAs). We conducted an observational single‐arm pilot trial to assess gut microbiota composition by 16S rRNA gene sequence analysis and to detect metabolite signatures by gas chromatography in stool samples from participants with HS prior to and 12 weeks after commencing adalimumab therapy. HS individuals that better responded to adalimumab treatment showed a shift in the composition and function of the gut microbiota with significantly increased SCFA acetate and propionate compared to age, gender and BMI‐matched healthy controls. A positive correlation was observed between propionate with *Prevotella sp* and *Faecalibacterium prausnitsii.* Increased SCFAs, changes in gut microbiota composition, function and metabolic profile following 12 weeks of adalimumab suggest that targeting SCFAs may be considered a potential biomarker to be evaluated as a complementary protective factor or as a diagnostically relevant signal in HS.

## 1 BACKGROUND

Hidradenitis suppurutiva is a chronic inflammatory skin disorder manifest in painful nodules, abscesses and purulent epithelialized tunnels with a predilection to flexural regions.^1^ It has an epidemiological association with inflammatory bowel disease, inflammatory arthropathy and metabolic syndrome.^1^ Dysregulation of both the cutaneous and gut microbiota composition has been observed in HS.^2^, ^3^, ^4^, ^5^, ^6^ In addition, recent work has identified numerous microbial products or metabolites that have emerged during disease or health to control physiology.^7^ For example, alterations of the gut and skin microbiota metabolic profile in individuals with HS are associated with enrichment of 5‐lipooxygenase metabolites^8^ and systemic alterations of tryptophan metabolism.^9^, ^10^ Particularly relevant examples include microbial metabolites such as short‐chain fatty acids (SCFAs) acetate, propionate and butyrate. They are produced from fibre fermentation by the gut microbiota and play essential roles in regulating inflammation, metabolism and gut homeostasis.^11^, ^12^

Promoting the stimulation of gut SCFA‐producing bacteria or increasing the production of SCFA acetate and butyrate play a critical role in tuning the human immune system with the potential to improve glycaemic control, as shown in T1D and T2D.^13^, ^14^ Likewise, in mouse models, targeted‐SCFA supplementation has shown to improve infection, colitis, type 1 and type 2 diabetes and facilitate anti‐cancer therapy.^13^, ^14^, ^15^, ^16^, ^17^, ^18^ Although anti‐inflammatory therapies are known to modulate the Psoriasis‐associated skin microbiota,^19^, ^20^ their effects on the microbiota biochemical profile, in particular on beneficial microbial metabolites such as SCFAs, remain unexplored. Here, we assessed whether adalimumab therapy impacts faecal microbiota and SCFAs in HS.

## 2 RESULTS

### 2.1 Participant characteristics

**FIGURE 1:**
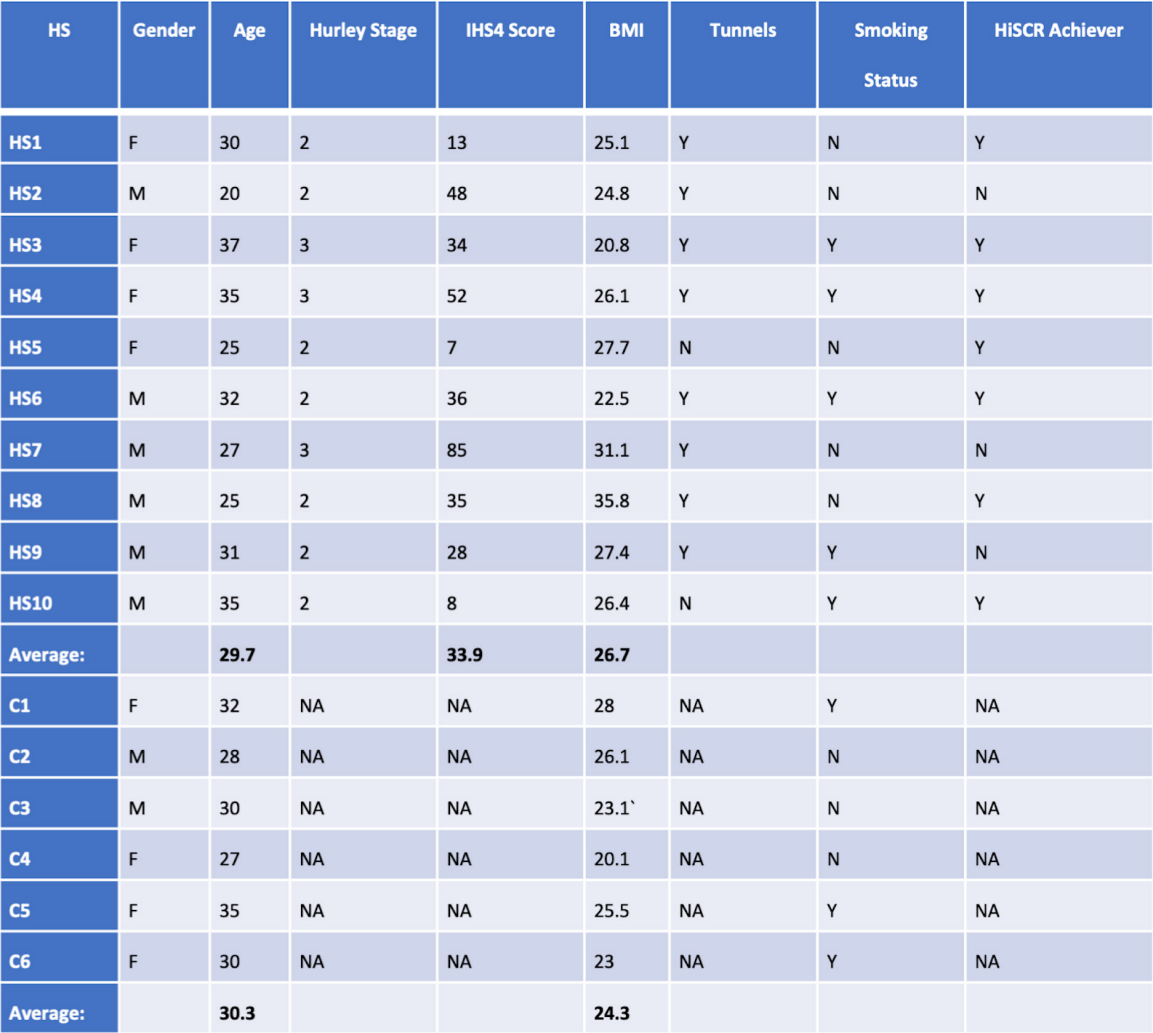
Demographics of the recruited participants. Ten hidradenitis suppurutiva (HS) subjects and 6 healthy controls were recruited for this study.

The demographic and disease‐specific variables included 10 participants with dermatologist‐diagnosed moderate to severe HS and six matched healthy controls presented in Figure 1. All individuals were over 18 years of age, not currently or previously treated with a biologic agent, and not treated with antibiotics for at least 8 weeks prior to enrolment. Also, none of the participants were treated with antibiotics during the study. The protocol for antibiotics was based on previous microbiota‐targeted studies in HS and T1D.^6^, ^13^ In addition, six matched healthy control participants without inflammatory skin disease were recruited from the same clinic. Individuals with HS received 12 weeks of subcutaneous adalimumab at standard FDA‐approved dosage (160 mg Week 0, 80 mg Week 2 and 40 mg every 2 weeks after that). Stool samples were collected at baseline (W0) and Week 12 (W12) after adalimumab in HS participants and healthy controls.

### 2.2 Adalimumab delivery is linked with changes in the gut microbiota composition and metabolic profile

**FIGURE 2:**
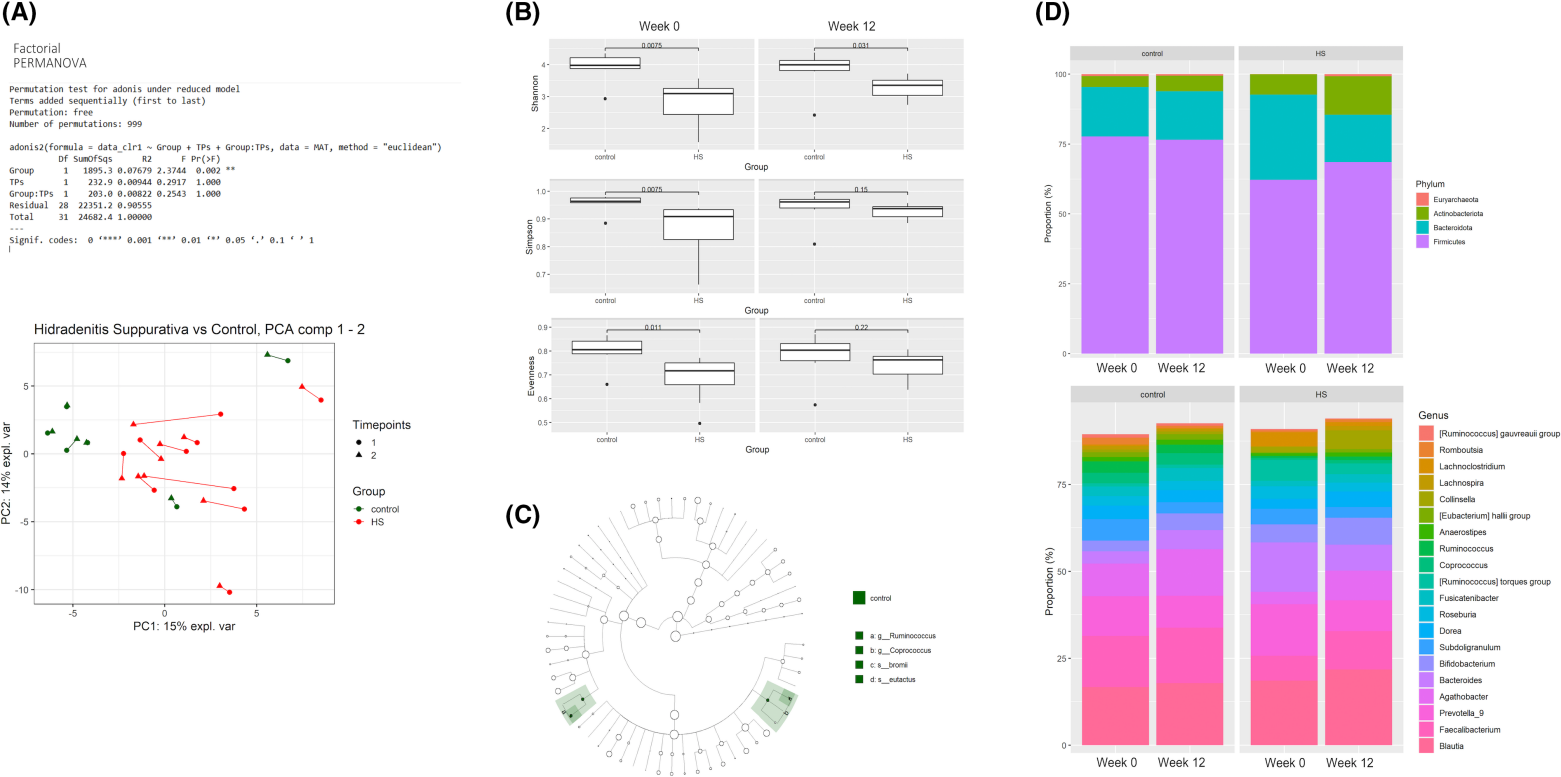
Composition and function of the gut microbiome are altered following adalimumab therapy. (A) Factorial PERMANOVA shows a significant difference in microbial composition between hidradenitis suppurutiva (HS) and control after accounting for timepoint difference *p* (0.002). Principal component analysis (PCA) comparing microbial species present at each time point. (B) Alpha diversity measured by Shanon, inverse Simpson index, and Evenness in healthy controls and HS participants at baseline (W0) and W12. (C) Cladogram demonstrating the significantly higher abundance of *Rumminocuccs bromii* and *Coprococcus eutactus* in healthy controls compared to individuals with HS at baseline. (D) A stronger phylum and genus level changes in the faecal microbial composition were detected in healthy controls and HS participants at W0 and W12.

A significant difference in the faecal microbial composition was detected between HS and controls after accounting for the timepoints and interaction (Factorial PERMANOVA, *R* ^2^ = 0.08, *p* = 0.002). Principal component analysis (PCA) illustrates a shift towards control samples. (Figure 2A). HS participants showed reduced alpha‐diversity (Pieolou's evenness, Shannon's richness index and Simpson's diversity index, *p* = 0.008–0.011) compared to healthy controls at W0, but the differences between healthy controls and HS at Week 12 were largely absent (Figure 2B). Differential abundance analysis using linear discriminant analysis Effect Size (LefSE) identified significantly higher in *Rumminococcus bromii* and *Coprococcus eutactus* in healthy controls versus HS (Figure 2C). Taxonomic composition of the microbiota at baseline (W0) between healthy controls and HS participants showed reduced members of the phylum Firmicutes and Euryarchaeota and increased proportion of Actinobacteria and Bacteroidetes (Figure 2D).

Treatment with 12 weeks of adalimumab at the FDA‐approved dosing regimen (160 mg at Week 0, 80 mg at Week 2, then 40 mg weekly from Week 4 to Week 12) showed a significant increase in alpha diversity similar to healthy controls (Shannon's richness index, *p* = 0.049; Figure 2B). HS faecal microbiota after 12 weeks of adalimumab therapy was more prevalent of the genus *Bifidobacterium*, *Bacteroides, Agathobacter, Prevotella_9*, *Faecalibacterium* and *Blautia* (Figure 2D). Faecal microbiota was no significant different in HS Clinical Response (HiSCR) achievers (≥50% reduction in inflammatory lesion count [abscesses + inflammatory nodules]) versus non‐achievers.

**FIGURE 3:**
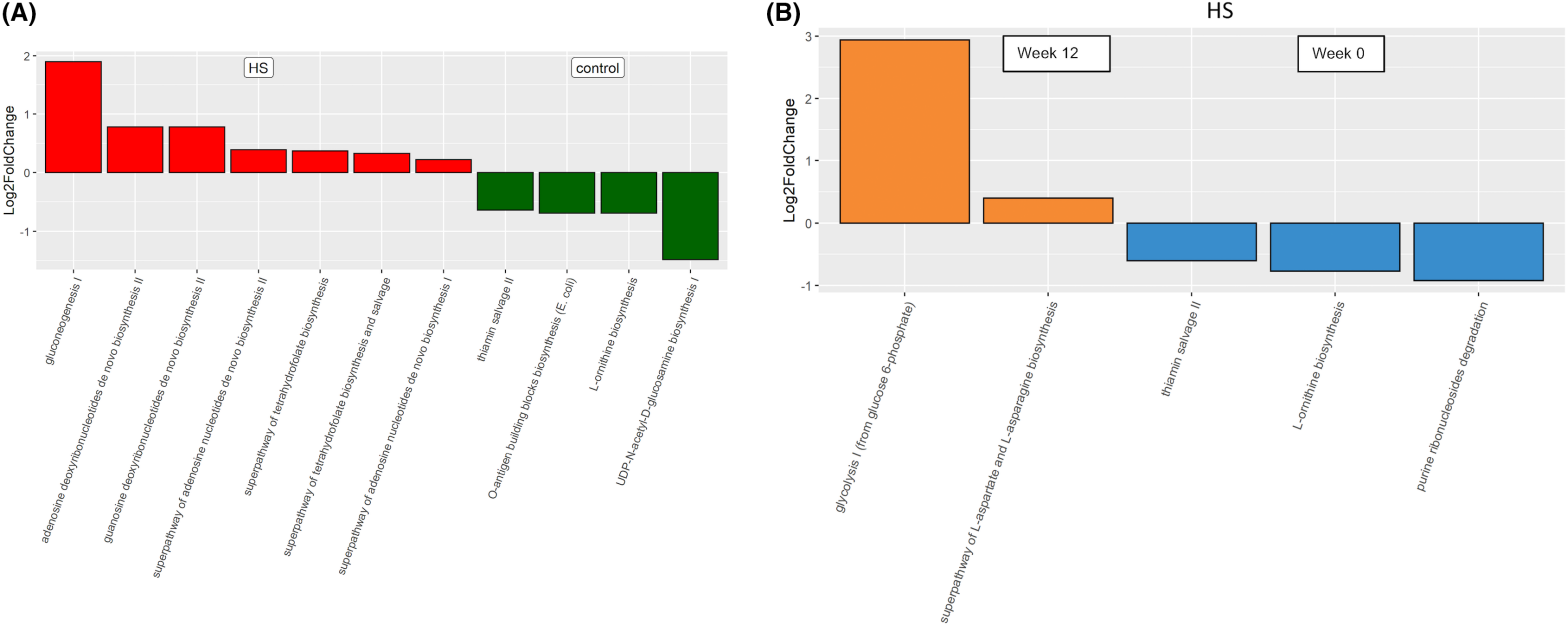
Adalimumab impact the faecal microbiota function and metabolic pathways. (A) An overall higher abundance of pathways related to carbohydrate biosynthesis and nucleoside and nucleotide biosynthesis were detected in hidradenitis suppurutiva (HS) than in healthy controls. When the comparison was made between time points (B), significant increases in the glycolysis I pathways at Week 12 compared with baseline were found in HS after adalimumab treatment. In contrast, no significant difference was seen in healthy controls. The features presented are significant by Benjamini–Hochberg (BH) adjusted *p* < 0.01.

PICRUSt2 functional bacterial profiles showed significant changes in carbohydrate energy production pathways between HS and control (Figure 3A). For instance, gluconeogenesis I and nucleotide biosynthesis were significantly elevated in HS, while UDP‐N‐acetyl‐D‐glucosamine biosynthesis I significantly changed in controls compared to HS participants. It is noteworthy that care must be taken when integrating this result as the pathways were imputed not based on the HS‐specific microbial genomes database. Subsequent shotgun metagenomics or targeted quantitative PCR is warranted to confirm this observation. Twelve weeks of adalimumab were followed by changes in metabolic pathways, including glycolysis I (Figure 3B).

### 2.3 Adalimumab treatment is followed by increased SCFAs in stools from HS participants

**FIGURE 4:**
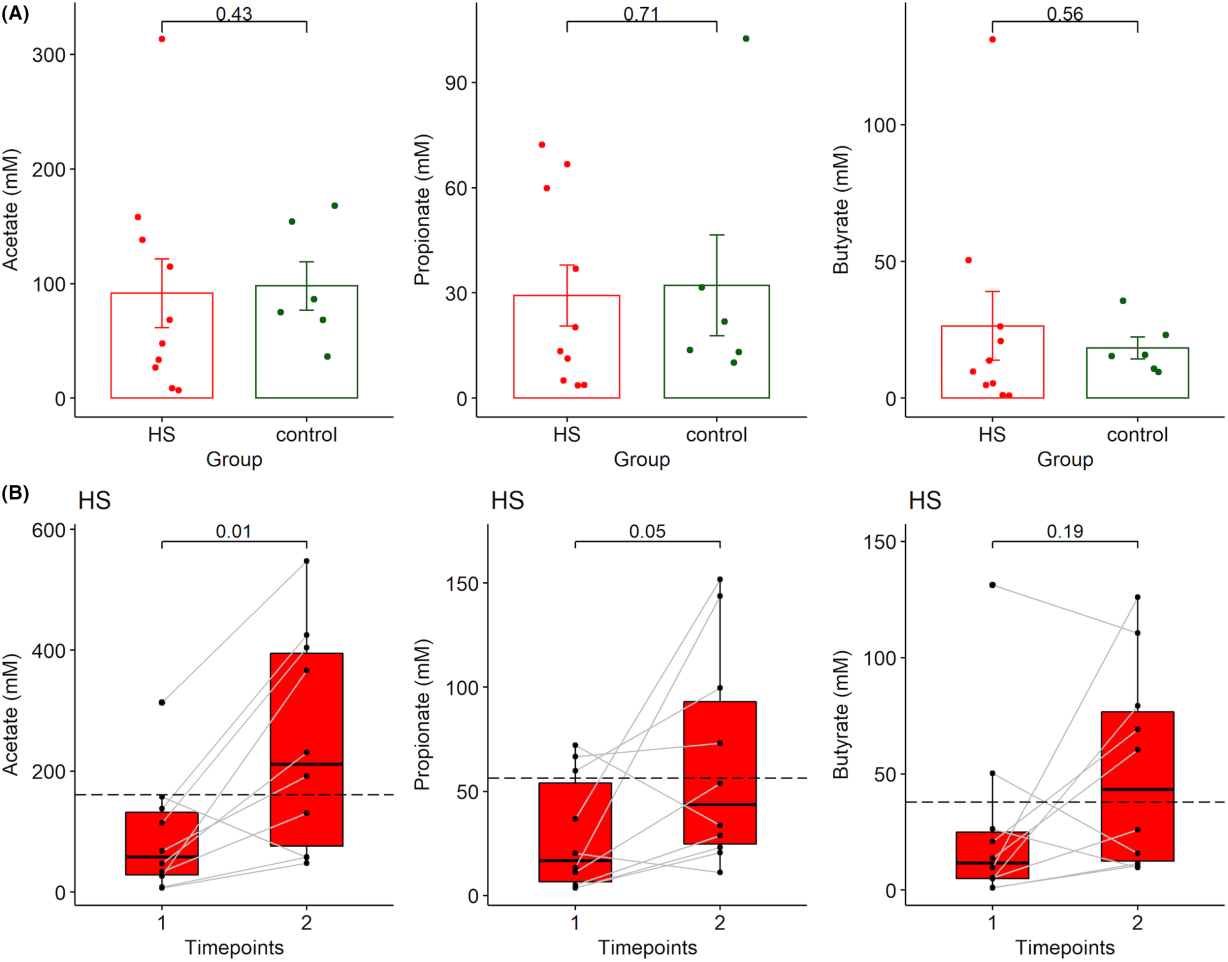
Increased concentration of short‐chain fatty acids in stool following adalimumab treatment. (A) Baseline concentrations of faecal SCFAs acetate, propionate and butyrate demonstrate no statistically significant difference between participants with HS and healthy controls. (*p* > 0.05 using Wilcoxon rank sum test with Bonferroni correction for multiple comparisons). (B) Significantly higher acetate and propionate were recorded after adalimumab intervention. The dotted line represents the mean of acetate, propionate and butyrate concentrations in healthy controls at W12, respectively.

There were no significant statistical changes in the faecal concentrations of SCFA acetate, propionate and butyrate at baseline between HS compared to age, gender and BMI‐matched healthy controls (Figure 4A). Stratification of HS participants by gender, smoking status, Hurley stage or presence of epithelialized tunnels did not identify any significant differences between groups regarding faecal acetate or butyrate at baseline using Wilcoxon rank sum test (Figure S1). Remarkably, increased faecal acetate and propionate were observed after 12 weeks of subcutaneous adalimumab therapy in HS, similar to healthy controls at W12 without treatment (Figure 4B). Increased stool butyrate concentrations were trending but not significant. Stratification of participants by gender, Hurley stage, HiSCR achievement and smoking status did not identify any significant differences between faecal SCFA at Week 12. Elevated faecal butyrate concentrations at Week 12 were associated with the presence of epithelialized draining tunnels (Figure S2).

**FIGURE 5:**
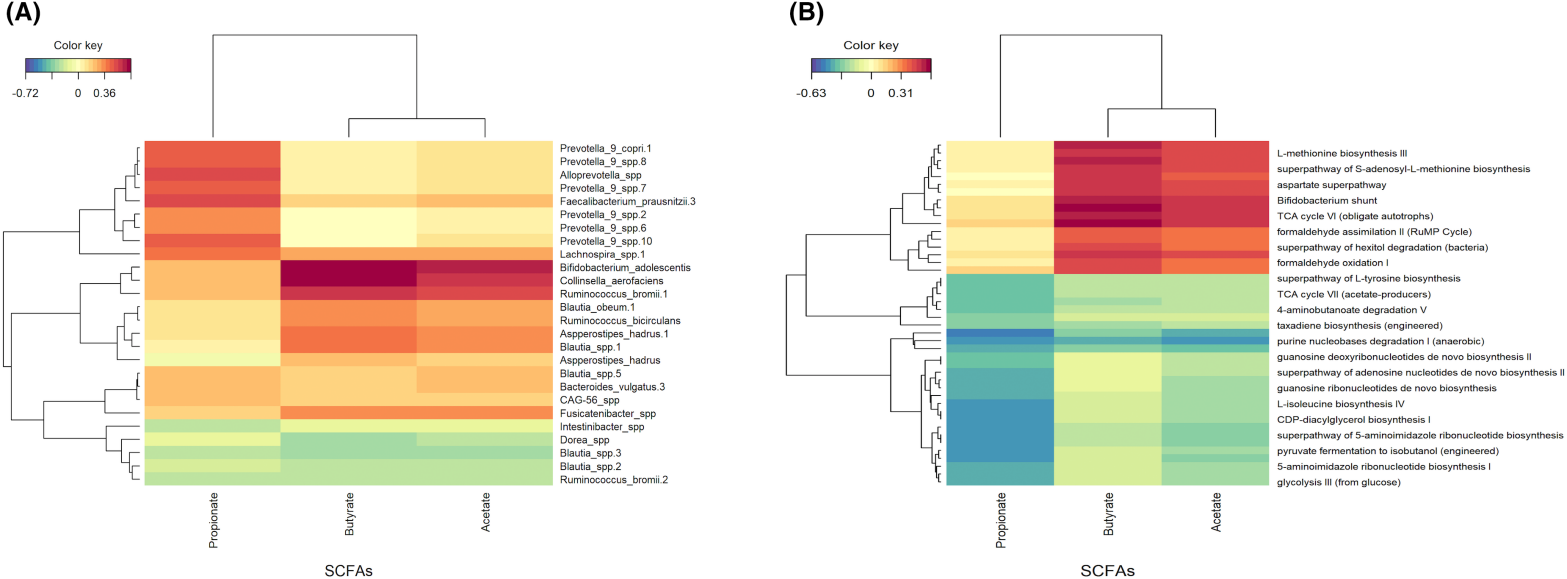
The relationship between the faecal microbiota and SCFAs in Hidradenitis Suppurativa (HS). Heatmap demonstrating the observed relationships between short‐chain fatty acid concentrations and observed genus (A) and metabolic pathways (B) across the two time points in individuals with HS.

A positive correlation was observed between propionate with *Prevotella sp* and *Faecalibacterium prausnitsii*. In addition, *Bifidobacterium adolescentis, Colinsella aerofaciens* and *Ruminococcus bromii* were positively correlated with butyrate and acetate (Figure 5A). The pathways driving the changes correlated with increased acetate and butyrate included bifidobacterium‐shunt, amino acid biosynthesis pathways (urea cycle and L citrulline biosynthesis), formaldehyde assimilation and oxidation pathways (Figure 5B). No association of faecal microbiome or SCFA alterations to clinical response was identified.

## 3 DISCUSSION

Adalimumab altered faecal microbiota composition, production of SCFAs, and metabolic function of participants with HS compared to age, gender and BMI‐matched healthy controls. While 16S ribosomal gene sequencing of the faecal microbiota in HS has recently been reported,^3^, ^6^ we demonstrate that the adalimumab therapy in HS shifts the microbiota and SCFAs production towards healthy controls. However, it was not associated with clinical response.

Inflammation in HS is heterogeneous, with a degree of systemic inflammation associated with the presence of epithelialized tunnels as measured by serum proteomics.^21^ No significant differences in SCFAs concentrations in stools were identified when stratified by clinical characteristics including gender, smoking and HiSCR achievement. The presence of tunnels is associated with reduced odds of clinical response to adalimumab therapy.^21^, ^22^ Significant differences in faecal butyrate concentrations were seen after 12 weeks of adalimumab therapy, with faecal butyrate higher in those individuals with epithelialized tunnels than those without (Figure S2). Theoretical explanations for the observed differences would include alterations in the active transport of butyrate across the gut epithelium (mediated by MCT1 and SMCT1 receptors) in individuals with tunnels or alterations in aryl hydrocarbon receptor signalling.^23^ Such mechanisms require clarification in future mechanistic studies.

Examining matched microbiota and SCFAs observations in HS is essential to understand the effect of microbiota alterations upon immunologically active metabolic mediators in the gut. Changes in the proportion of *Prevotella spp* and propionate concentrations in HS after adalimumab treatment were consistent with previous documentation of *Prevotella spp* being important in disease activity of HS^24^ and consistent with previous cutaneous microbiota data in HS (Ring et al., 2017). In addition, reductions in SCFAs produced by *Bifidobacteria* and *Ruminococcus* have been associated with the accumulation of visceral adiposity^25^ and defective mucosal host defense,^26^ both characteristics associated with HS.^1^

Microbial carbohydrate energy production pathways such as gluconeogenesis I, nucleotide biosynthesis were significantly elevated in HS, with UDP‐N‐acetyl‐D‐glucosamine biosynthesis I significantly changed in controls. The UDP‐N‐acetyl‐D‐glucosamine biosynthesis pathway is also active during yeast metabolism and is highly associated with severe HS.^27^ Twelve weeks of adalimumab were associated with changes in metabolic pathways, including glycolysis I. Reductions in purine and L‐ornithine biosynthesis were seen at W0 in HS compared to W12. It is worthy to note that we use PICRUSt2 as a bioinformatic tool for predicting functional content from 16S rRNA gene sequence data, which uses phylogeny to predict genomic content. Although it has been used in other microbiota‐targeted clinical trials,^28^, ^29^ it is not without limitations and used on the assumption that it can adequately reconstruct metagenomic data. Therefore, the results provided only a direction for subsequent gene expression studies.

Restoration of the gut microbiota suppresses the clinical signs of atopic dermatitis (AD) associated with increased faecal SCFAs, induced Tregs, reduced IgE levels and the numbers of mast cells, eosinophils and basophils.^30^ It is known that adalimumab alters the faecal microbiota in rheumatoid arthritis and Crohn's disease.^31^, ^32^ Overall, our data are the first to demonstrate that adalimumab modulates the biochemical profile of the gut microbiota by increasing the production of faecal SCFAs in HS. Indeed, the pathways driving the changes correlated with increased acetate and butyrate included bifidobacterium‐shunt, amino acid biosynthesis pathways (urea cycle and L citrulline biosynthesis), formaldehyde assimilation and oxidation pathways. Interestingly, similar pathways modulated by adalimumab were found in individuals with T1D after 6 weeks of consuming a dietary supplement that enhanced circulating and faecal SCFAs.^13^

Positive correlations observed between the SCFAs and *Prevotella sp*, *Faecalibacterium prausnitsii*, *Bifidobacterium adolescentis, Colinsella aerofaciens* and *Ruminococcus bromii* are well documented.^33^, ^34^, ^35^ In addition, changes in the proportion of *Prevotella spp* and propionate concentrations in HS after adalimumab treatment are consistent with previous documentation of *Prevotella spp* being important in disease activity of HS.^24^ Interestingly, reductions in SCFA produced by *Bifidobacteria* and *Ruminococcus* have been associated with accumulation of visceral adiposity^25^ and defective mucosal host defense,^26^ both characteristics associated with HS.^1^

### 3.1 Limitations of the study

The study included a small sample size and limited follow‐up of the study. Without a placebo control group, this study could not determine whether any of the changes observed are caused by the therapy or only associative. A follow‐up placebo‐controlled trial powered is needed to determine causal effects from adalimumab through the production of anti‐inflammatory SCFAs in a larger study and longer follow‐up period of intervention to better determine any impact on clinical response.

## 4 METHODS

### 4.1 Study population

Adult participants with a confirmed clinical diagnosis of HS made by a consultant dermatologist were invited to participate in the study. The South Western Sydney Local Health District Human Research Ethics Committee provided ethical approval for this study (Approval Number 2019/ETH09974). Written informed consent was obtained from all participants in line with the Declaration of Helsinki. Exclusion criteria included participants under 18 years of age, current or previous treatment with a biologic agent, topical or oral anti‐microbial therapy. Clinical data including age, gender, BMI, smoking status, height, weight, disease severity as measured by Hurley Staging, abscess, nodule and tunnel counts were recorded at baseline. All HiSCRs were performed by the same clinician experienced in HS clinical scoring. Two individuals achieved HiSCR 75, but none achieved HiSCR 100. Healthy control participants were recruited from the general population and were age, gender and BMI matched to HS participants.

### 4.2 Adalimumab therapy

Subcutaneous adalimumab therapy was provided at the FDA‐approved dose (160 mg Week 0, 80 mg Week 2, and 40 mg Week 4, 6, 8, 10 and 12). All participants underwent appropriate screening for HIV, hepatitis B, hepatitis C and quantiferon gold testing for latent tuberculosis prior to enrolment in the study. All participants continued on adalimumab therapy as a standard of care after completing involvement in the trial. Healthy controls were not exposed to adalimumab therapy.

### 4.3 Sample collection

Faecal samples (~1 g) from participants with HS (*n* = 6) were collected at baseline (prior to the first dose of adalimumab) and Week 12 of adalimumab therapy. Faecal samples from healthy controls (*n* = 6) were taken at baseline and Week 12 time points. All faecal samples were collected at the time of clinical examination. Faecal samples were immediately frozen at −80°C until processing.

### 4.4 Short‐chain fatty acid analysis

Acetate, propionate and butyrate were measured in duplicates by gas chromatography after liquid–liquid extraction, as reported previously (Marino et al., 2017). Stool samples were mixed with internal standards solution and centrifuged. One hundred microliters of supernatant was filtered through the 100 μm mesh filters and transferred to the glass insert GC vials. Samples were analysed on an Agilent 7890A gas chromatograph (Agilent Technologies). Peaks were detected with a flame ionization detector at 210°C and identified and quantitated against calibration standards over the range of 0 to 400 mM. Intra‐assay CVs were 14.2%, 11.8% and 10.3% for acetate, propionate and butyrate, respectively.

### 4.5 16S rRNA gene sequence analysis

Total DNA was extracted using the QIAamp DNA stool mini kit (Qiagen). Briefly, the stool was homogenized in Buffer ASL using a tissue disperser (T 10 basic ULTRA‐TURRAX®, IKA) for 1 min and processed following the manufacturer's protocol. Amplification of DNA samples was performed by Micromon, Monash University, by targeting the V3‐V4 region of bacterial 16S rRNA gene using forward primer 5′‐ CCTACGGGNGGCWGCAG‐3′ and reverse primer 5’‐GACTACHVGGGTATCTAATCC‐3′. The PCR reaction was carried out in 25 μl volumes containing 5 ng/μl DNA template, 1 μM of each primer and 12.5 μl of 2X KAPA HiFi HotStart ReadyMix (Kapa Biosystems). The following PCR conditions were used: The following PCR conditions were used: initial denaturation at 95°C for 3 min, followed by 25 cycles consisting of denaturation (95°C for 30 s), annealing (55°C for 30 s), and extension (72°C for 30 s) and a final extension step at 72°C for 5 min. PCR products were purified using AMPure XP beads (Beckman Coulter) and sequenced using Illumina Miseq sequencer. For microbiota bioinformatics, a total of 3 161 460 raw paired‐end reads were obtained from the Illumina MiSeq amplicon sequencing, targeting the V3‐4 region of the 16S rRNA gene. The sequences were denoised, quality filtered and defined into Amplicon Sequence Variants (ASVs) using the dada2 R package.^36^ The final dataset consisted of 1 463 912 merged contigs. The taxonomy was assigned using the Silva 138.1 prokaryotic SSU taxonomic training data. Both taxonomy and abundance tables were imported into phyloseq R package^37^ for further analysis. ASVs with relative abundance <1% and present in <5% of samples were removed to reduce background noise. A total of 110 ASVs were retained after the filter, and alpha diversity indices such as Shannon, Simpson and Pielou's J (evenness) were calculated.

### 4.6 Statistical analysis

The statistical significance of the alpha diversity indices between different timepoints was derived using Wilcoxon signed rank test. The same test was also conducted to compare the expression of SCFAs across timepoints. For beta diversity, the data were first transformed into Aitchison distance, and the differences between the bacterial composition across timepoints and subject groups (i.e. control and HS) were compared using Factorial, Repeated Measure and Pairwise PERMANOVA separately (vegan R and pairwiseAdonis R).^38^, ^39^ In addition, differentially abundant taxa were identified using LEfSE^40^ while the differentially abundant PICRUSt2 pathways were inferred using LIMMA R Package^41^ adjusting for multiple corrections using the Benjamini–Hochberg approach. The associations between the bacterial taxa with SCFAs and pathways with SCFAs were established using the sparse partial least square analysis (sPLS) implemented by the mixOmics R package.^42^ Statistical Analysis for Clinical Associations between clinical characteristics and clinical outcomes (HiSCR) was completed using the Wilcoxon rank‐sum test.^43^ Adjustment was made using the Bonferroni method for multiple comparisons. *p* < 0.05 was considered statistically significant. All statistical analysis for clinical associations was undertaken using Prism (8.4.2).

## AUTHOR CONTRIBUTIONS

Conceptualization: GDC, JAW, JWF, EMM. Data Curation: SAS, AT, JWF, EMM. Formal Analysis: EMM, JWF, SB, CCW. Funding Acquisition: JAW, GDC, Investigation: SAS, ATT, SB, CCW. Methodology: JAW, GDC, JWF, EMM. Project Administration: JAW, GDC, JWF, EMM. Resources: JAW, GDC, EMM. Software: JWF, EMM, CCW. Supervision: GDC, JAW, JWF, EMM. Validation: JWF, EMM, CCW. Visualization: JWF, CCW, EMM. Writing–Original Draft: JWF, EMM. Writing Review and Editing: SDS, AT, JAW, GDC, SB, CCW, EMM, JWF.

## FUNDING INFORMATION

Funding was provided via an unrestricted educational grant from Sun Pharma. Sun Pharma had no role in collecting, analysing or interpreting data, writing the report or deciding to submit the report for publication.

## CONFLICT OF INTEREST

JWF has conducted advisory work for Janssen, Boehringer‐Ingelheim, Pfizer, Kyowa Kirin, LEO Pharma, Regeneron, Chemocentryx, Abbvie and UCB. He has participated in trials for Pfizer, UCB, Boehringer‐Ingelheim, Eli Lilly and CSL, and received research support from Ortho Dermatologics and Sun Pharma. CCW provides data science consultation for Asian Microbiome Library, Singapore.

## Supporting information


**Figure S1.** No significant difference was seen between faecal SCFA concentrations in participants with HS at baseline when stratified by the presence of epithelialized tunnels or smoking status (Assessed using Wilcoxon Rank Sum test with Bonferroni Adjustment for multiple comparisons).

**Figure S2.** Significant elevation in faecal Butyrate found at W12 of adalimumab therapy in individuals with epithelialized tunnels compared with those without epithelialized tunnels (*p* < 0.05; Assessed using Wilcoxon Rank Sum test with Bonferroni Adjustment for multiple comparisons).

## Data Availability

16S dataset is deposited in Bioproject http://www.ncbi.nlm.nih.gov/bioproject/804484 (accession code PRJNA804484)
