# Supplementary figures and images for: Adalimumab therapy is associated with increased faecal short chain fatty acids in hidradenitis suppurativa
Source: Exp Dermatol. 2022 Aug 30;31(12):1872–80. doi: 10.1111/exd.14665 (PMC10087920; doi:10.1111/exd.14665)

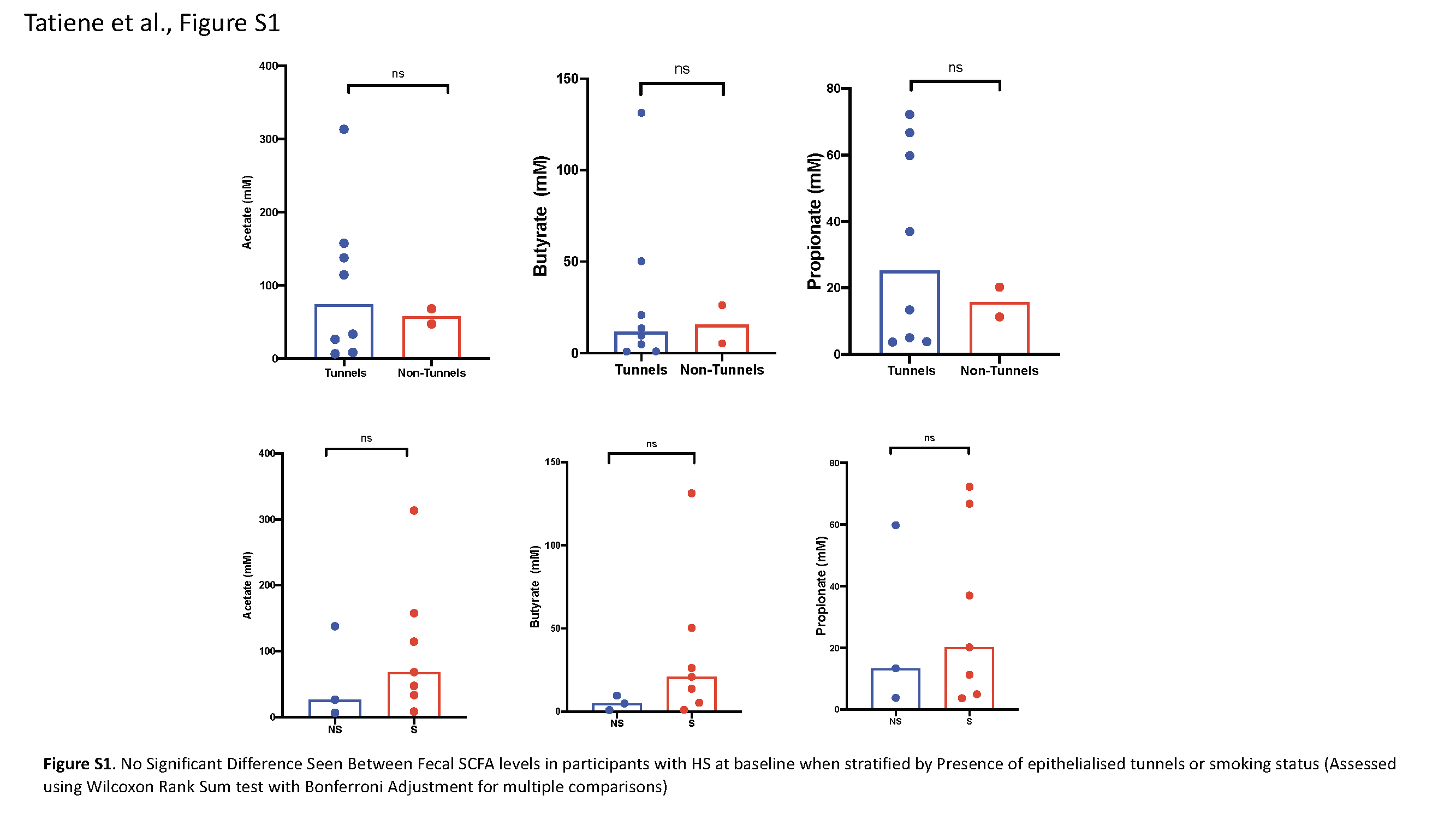

Supplement: Supplementary file 1 — Figure S1. No significant difference was seen between faecal SCFA concentrations in participants with HS at baseline when stratified by the presence of epithelialized tunnels or smoking status (Assessed using Wilcoxon Rank Sum test with Bonferroni Adjustment for multiple comparisons). [file EXD-31-1872-s001.tiff]

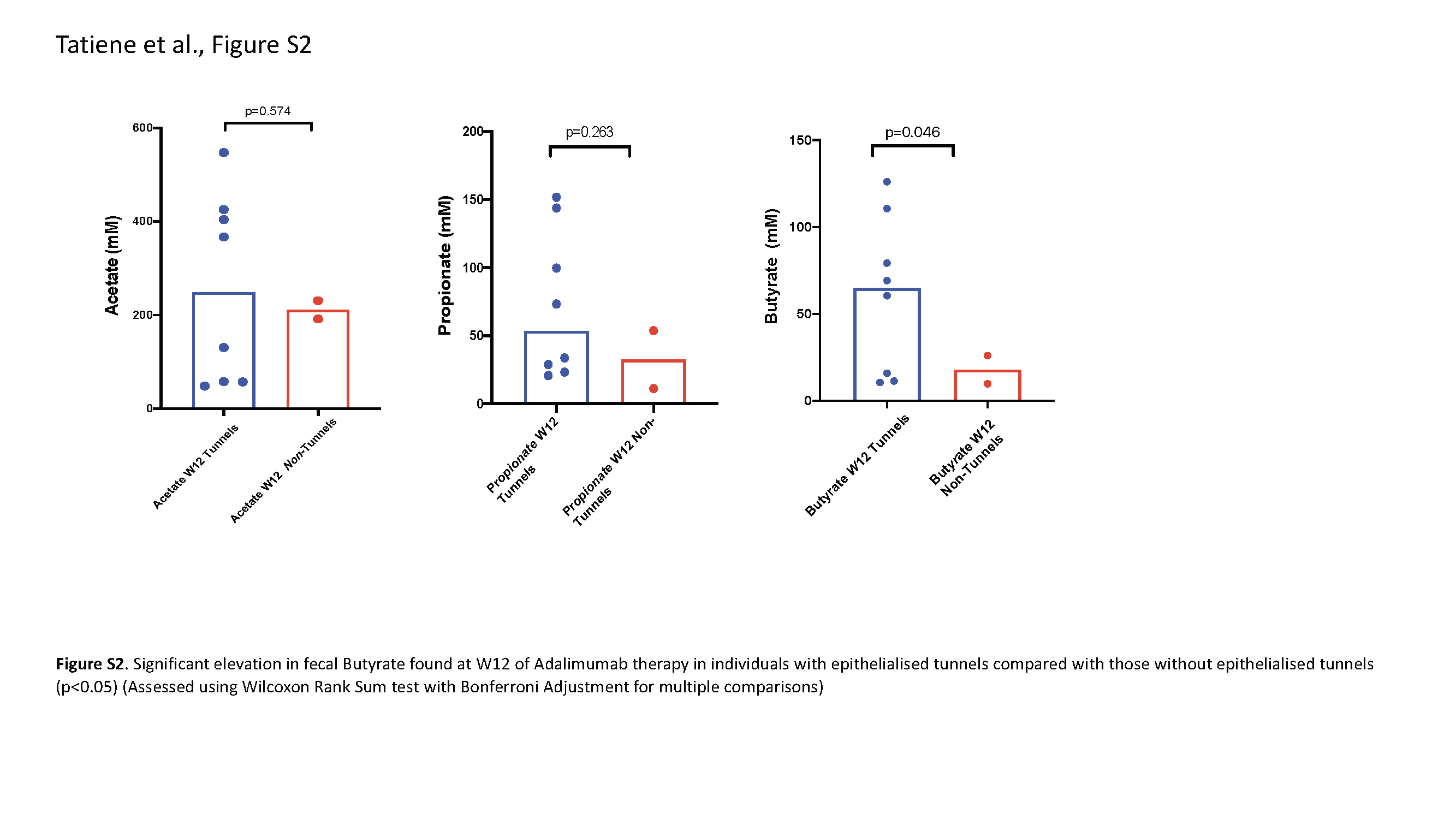

Supplement: Supplementary file 2 — Figure S2. Significant elevation in faecal Butyrate found at W12 of adalimumab therapy in individuals with epithelialized tunnels compared with those without epithelialized tunnels (p < 0.05; Assessed using Wilcoxon Rank Sum test with Bonferroni Adjustment for multiple comparisons). [file EXD-31-1872-s002.tiff]
